# Correlation among the structural, electric and magnetic properties of Al^3+^ substituted Ni–Zn–Co ferrites

**DOI:** 10.1039/d1ra09354a

**Published:** 2022-05-18

**Authors:** Nusrat Jahan, M. N. I. Khan, M. R. Hasan, M. S. Bashar, A. Islam, M. K. Alam, M. A. Hakim, J. I. Khandaker

**Affiliations:** Department of Physics, Jahangirnagar University Savar Dhaka 1342 Bangladesh nusrat1974@yahoo.co.uk hoqueh2003@yahoo.com jahir-nanophysics@juniv.edu; Department of Physics, American International University Bangladesh (AIUB) Dhaka 1229 Bangladesh; Materials Science Division, Atomic Energy Centre Dhaka 1000 Bangladesh ni_khan77@yahoo.com razibhasan68@gmail.com; Sustainable Energy Technology, Institute of Fuel Research & Development, BCSIR, Ministry of Science & Technology Bangladesh bashar@agni.com; Department of Physics, Magura Govt. Mahila College Magura Bangladesh amin418847@gmail.com; Department of Physics, Materials Science Lab, Bangladesh University of Engineering & Technology Dhaka 1000 Bangladesh khurshedphy@phy.buet.ac.bd; Department of Glass and Ceramic Engineering, Bangladesh University of Engineering and Technology (BUET) Dhaka 1000 Bangladesh hakim.akm@gmail.com

## Abstract

This study explored the structural, electrical, and magnetic properties of diamagnetic aluminium (Al^3+^) substituted nickel-zinc-cobalt (Ni–Zn–Co) mixed spinel ferrites, though the research on this area is in the infancy stage. Single-phase cubic spinel structures with the *Fd*3̄*m* space group of the synthesized Ni_0.4_Zn_0.35_Co_0.25_Fe_(2−*x*)_Al_*x*_O_4_ (0 ≤ *x* ≤ 0.12) ferrite samples were confirmed by X-ray diffraction (XRD) analysis. The average particle size ranged from 0.67 to 0.39 μm. Selected area electron diffraction (SAED) patterns were indexed according to the space group *Fd*3*m*, representing the particle's crystallinity. The optical band gaps ranged from 4.784 eV to 4.766 eV. Frequency-dependent dielectric constants and ac conductivity measurement suggested that the prepared ferrites were highly resistive. Relaxation times were reduced to a low value from 45.45 μs to 1.54 μs with the composition *x*. The Curie temperatures (*T*_c_) were 615–623 K for all samples. Real part permeabilities (*μ*^/^) were relatively stable up to an extended frequency range of 10^6^ Hz with relative quality factors (RQF) of around 10^3^. Tuning of the properties indicates that the fabricated ferrites may be promising for high-frequency electronic devices.

## Abstract 2

This study explored the structural, electrical, and magnetic properties of diamagnetic aluminium (Al^3+^) substituted nickel–zinc–cobalt (Ni–Zn–Co) mixed spinel ferrites, though the research on this area is in the infancy stage.

## Graphical abstract

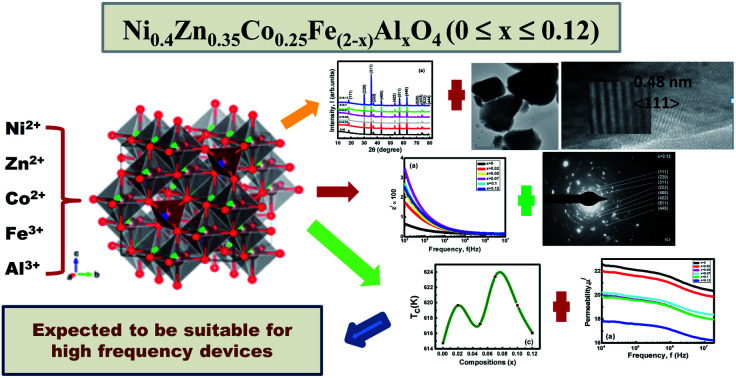

## 1. Introduction

Recently, ferrimagnetic oxides, or ferrites, have risen to significance due to their intelligent designs of chemical compositions and crystal structures with suitable characteristics.^1^ Spinel-based cubic ferrite possesses dual electric and magnetic properties depending on its structural compositions, cationic arrangements, sintering temperature, and fabrication methodology.^1–4^ Several synthesis techniques, including solid-state reactions, sol–gel, hydrothermal, ball milling, co-precipitation, *etc.*, have been established over the years to fabricate ferrites of different sizes and shapes.^5–8^ Ferrites are ideal for high-quality multifaceted applications like home and industrial electronic devices, biomedical devices, catalysts, and EMI shielding in absorber materials because of their relatively high dielectric/magnetic loss, excellent chemical stability, and low cost.^9^ The features like the high permeability in ferrite make it an attractive candidate for wide-ranging applications such as microwaves, gas sensing, multilayer chip inductor, *etc.*^4^ Moreover, high Curie temperatures and moderate magnetic losses in the kHz range make ferrites promising for constructing heating inductors in the catalytic chemical reactors.^10^ Other than that, the function of ferrites for microwave heating and magnetic separation in chemical reactors offers a new scientific platform for hi-tech integration.^10^

Mn–Zn ferrite has the largest production and market share among ferrimagnetic oxides due to their outstanding efficiency under the ac field at frequencies until 1 MHz.^11^ However, for the growing electronics, telecommunication, and power applications at higher frequencies, Ni–Zn ferrites are functioning remarkably.^11^ These materials retain higher electrical resistivity that certifies sufficiently low power losses and high magnetic responses in this high-frequency zone.^11^ Ni–Zn ferrite is generally touted among the spinel ferrites for its applications in magnetic, magnetoptical, and magnetodielectric devices.^12^ Besides, in recent years, Ni–Zn ferrites have paid attention to microwave applications, high-frequency devices like multilayer chip inductors, and electromagnetic interference filters because of their extraordinary magnetic properties and dielectric values while revealed to high frequencies.^13,14^

It was reported in the literature that the characteristics of spinel ferrite are tuned when doped with divalent and trivalent cations. It was also informed that substituting tetravalent ions improves ferrite's electromagnetic properties.^15^ Hosting Co in Ni–Zn ferrites can extend the operational band at higher frequencies, ranging until cut off.^11^ Thus, the Ni–Zn–Co ferrites can alter themselves to work effectively for devices that function above 1 MHz frequency.^11^

However, Das and Singh^16^ explored the structural, magnetic, and dielectric properties of Cu-doped Ni–Zn ferrites.^13^ They stated that the coercivity and saturation magnetization of Ni–Zn ferrites enhanced by substituting Cu content.^13^ Additionally, introducing Co^2+^ ions in Ni–Zn ferrites improves the strain sensitivity and resistive properties with a reduction in dielectric loss.^17^ Substitution of Zn^2+^ in Co ferrites leads to enhancement of dielectric and magnetic properties.^17^ Li *et al.*^18^ understood that charge carrier reorganization induced by magnetic field enhanced electron–electron correlation, increasing the electron-hopping between cations leads to resistance declination. Atif *et al.*^19^ investigated the magnetic and dielectric properties in Zn doped cobalt ferrite. They recorded the dielectric constant at low frequencies, implying its use as a stress sensor.^8^ Yaseneva *et al.*^20^ found that the Zn substitution caused a reduction of *T*_c_.^8^ Li *et al.*^21^ stated that the partial inversion of cations (Ni, Fe) in nickel ferrite reallocated them inside the sublattices to generate magnetic hardening. Furthermore, adding Ni^2+^ ions in Co ferrites hindered the grain formation, resulting in low surface roughness.^17^ Ni–Zn–Co ferrites are an exciting system due to their wide range of biomedical and microwave devices.^22^

The substitution of nonmagnetic Al^3+^ in the nickel ferrites declined the charge transfer mechanism between Fe^3+^ and Fe^2+^ ions. Thus, a composition with less dielectric loss and high resistivity was found.^3^ These unique properties are suitable for manufacturing radio and microwave devices operating at L, S, and C bands.^23^ Other than that, Al^3+^ ions addition in Ni-ferrite inhibits grain growth and improves the ferrites' mechanical strength.^24^ Besides, Al–O–Al areas hinder grain growth after Al^3+^ ion substitution in Ni–Zn–Co ferrites.^1^ The substitution of Al^3+^ ions in nickel ferrites reduced the ferrites' coercivity and tuned the compositions suitable for high-frequency applications.^23,24^ Substitution of Al^3+^ ions in the cobalt ferrites reduces magnetic hardness and enhances electrical resistivity. Al^3+^ substituted cobalt ferrites' electrical and magnetic properties were perfect for power transformer core materials in communication applications.^23^

In the present work Al^3+^ doped Ni_0.4_Zn_0.35_Co_0.25_Fe_(2−*x*)_Al_*x*_O_4_ (0 ≤ *x* ≤ 0.12) mixed ferrites were fabricated through conventional ceramic technique. Ni_0.4_Zn_0.35_Co_0.25_Fe_(2−*x*)_Al_*x*_O_4_ (0 ≤ *x* ≤ 0.12) was a newly studied composition.^1^ However, these studied compositions' optical, electrical, and magnetic properties remain unexplored. Thus, the newly made ferrites studies appear intriguing to see how doping changes properties that make them appropriate to high-frequency devices.

## 2. Experimental

### 2.1 Sample preparation

Magnetic cubic spinels with chemical compositions Ni_0.4_Zn_0.35_Co_0.25_Fe_(2−*x*)_Al_*x*_O_4_ (0 ≤ *x* ≤ 0.12) prepared by the conventional ceramic technique. Merck, Germany's high purity (99.95–99.98%) raw materials (Ni_2_O_3_, ZnO, Co_3_O_4_, Fe_2_O_3_, and Al_2_O_3_) were weighted stoichiometric ratio. Oxides were hand-milled carefully for 6 h by using an agate mortar and pestle.^25^ The homogeneous mixtures were mixed with deionized (DI) water and compressed (5 kN) into circular tablets. The tablets were pre-sintered at 800 °C for 4 h at a heating rate of 5 °C min^−1^. Tablets were crushed and grounded again for 2 h. The grounded fine powders mingled with polyvinyl alcohol (5% PVA) as a binder and pressed at a pressure of 10 kN and 15 kN to prepare the pellet (8.4 mm) and toroid (11.8 mm) shape samples. Finally, the samples were sintered at a temperature of 1200 °C for 5 h in air and allowed to cool down gradually inside the furnace at room temperature. The prepared ferrites were ready for characterizations. The mechanism of the chemical synthesis in equation form is shown below:Ni_2_O_3_ + ZnO + Co_3_O_4_ + Fe_2_O_3_ + Al_2_O_3_ → Ni_0.4_Zn_0.35_Co_0.25_Fe_(2−*x*)_Al_*x*_O_4_ (0 ≤ *x* ≤ 0.12)

### 2.2 Characterizations

Single-phase structural identifications of the studied ferrites were conducted by the Fast Detector X-ray diffractometer (Model: SmartLab SE, Rigaku Corporation, Japan) using Cu-K_α_ radiation of wavelengths 1.54059 Å and 1.54441 Å at room temperature. Energy-Dispersive X-ray spectra and surface morphology of the typical samples with *x* = 0−*x* = 0.12 were directly examined by the high-resolution Field Emission Scanning Electron Microscopy (FE-SEM) (JEOL, JSM-7610F). Duel Beam UV-VIS spectrophotometer (Model no: U-2900, Hitachi High-Tech Corporation, Tokyo, Japan) recorded absorption spectra data. The bandgap of the samples was measured from the absorption spectra of wavenumbers 190 to 1100 cm^−1^. Dielectric and permeability measurements were carried out through the Impedance analyzer (Wayne Kerr 6500B). The temperature dependence magnetizations and Curie's temperature were measured using Quantum Design's physical properties measurement system (PPMS).

## 3. Results and discussion

### 3.1 X-ray diffraction analysis

**Fig. 1:**
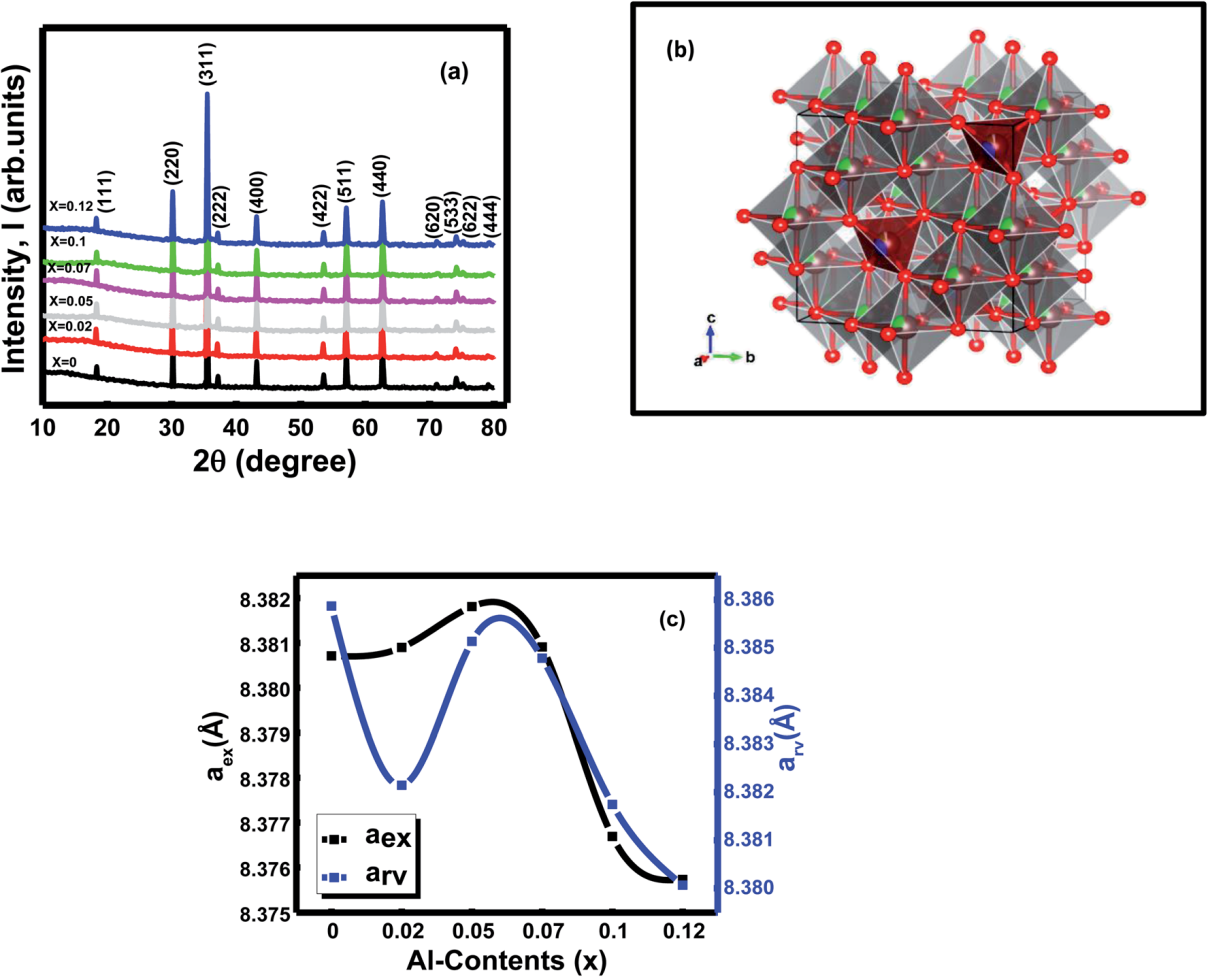
(a) X-ray patterns of Ni_0.4_Zn_0.35_Co_0.25_Fe_(2−*x*)_Al_*x*_O_4_ (0 ≤ *x* ≤ 0.12) ferrites (b) crystal structure of composition *x* = 0 and (c) variation of lattice constants (*a*_e*x*p_ and *a*_rv_) with compositions *x*.

The X-ray diffraction spectra of all the synthesized ferrites were observed at room temperature (Fig. 1a). The X-ray pattern of the studied samples confirmed single-phase cubic spinels' formation without any secondary phase and impurities. The peaks indexed as (111), (220), (311), (222), (400), (422), (511), (440), (620), (533), (622), (444) according to the standard JCPDS card no. 52-0277and JCPDS card no. 08-0234.^1^ Rietveld refinement of the X-ray diffraction data using FULLPROF suite program^26^ brought from the Jahan *et al.* 2021(1). Refinement of the XRD data also confirmed the formation of Ni_0.4_Zn_0.35_Co_0.25_Fe_(2−*x*)_Al_*x*_O_4_ (0 ≤ *x* ≤ 0.12) spinel ferrite system with the space group *Fd*3̄*m* (227) (27 22). The metal ions (Ni^2+^, Co^2+^, Zn^2+^, Fe^3+^, and Al^3+^) occupied the tetrahedral (8a) and octahedral (16d) crystallographic sites according to their site preferences.^27^ Rietveld's refinement of XRD data proceeded by refining the lattice parameters, background coefficient, and profile parameters first, followed by the refinement of Wyckoff positions and occupancy of the existing cations and anions. Table 1 represents the Wyckoff site with atomic coordinates (*x*, *y*, *z*) and occupancy of the ions that are present inside the compositions. The profile parameters found from the fitted patterns for all the samples are given in Jahan *et al.* 2021(1). The samples' noisy XRD data and the strongest reflections' intensities to the background ratios were very low, and slightly high values of profile factor (*R*_p_) were obtained.^1^ The chi2 values range from 1.02 to 1.19,^1^ indicating refinements are reliable in the goodness of fitting approach towards the samples' specific structures. The crystal structure of the composition *x* = 0 drew through the Rietveld refinement XRD parameters CIF file and is shown in Fig. 1b.

**Table 1:** Wyckoff sites (WS), atomic coordinates (*x*, *y*, *z*), and occupancy (Occ) of existing ions of Al substituted Ni–Zn–Co ferrites

Ions	WS	*x* = 0		*x* = 0.02		*x* = 0.05		*x* = 0.07		*x* = 0.1		*x* = 0.12	
		*x* = *y* = *z*	Occ	*x* = *y* = *z*	Occ	*x* = *y* = *z*	Occ	*x* = *y* = *z*	Occ	*x* = *y* = *z*	Occ	*x* = *y* = *z*	Occ
O	32(e)	0.2552	0.1667	0.2565	0.1667	0.2542	0.1667	0.2572	0.1667	0.2566	0.1667	0.2569	0.1667
FeT	8(a)	0.125	0.2125	0.125	0.0275	0.125	0.0283	0.125	0.0238	0.125	0.0283	0.125	0.0254
FeO	16(d)	0.500	0.0621	0.500	0.055	0.500	0.0529	0.500	0.0567	0.500	0.0529	0.500	0.0529
AlT	8(a)	0.125	—	0.125	—	0.125	—	0.125	—	0.125	—	0.125	0.0017
AlO	16(d)	0.500	—	0.500	0.0008	0.500	0.0021	0.500	0.0029	0.500	0.0038	0.500	0.0033
NiO	16(d)	0.500	0.0168	0.500	0.0168	0.500	0.0168	0.500	0.0168	0.500	0.0168	0.500	0.0168
ZnT	8(a)	0.125	0.0146	0.125	0.0142	0.125	0.0133	0.125	0.0146	0.125	0.0133	0.125	0.0146
ZnO	16(d)	0.500	—	0.500	0.0042	0.500	0.0013	0.500	—	0.500	0.0013	0.500	—
CoT	8(a)	0.125	0.0058	0.125	—	0.125	—	0.125	0.0033	0.125	—	0.125	—
CoO	16(d)	0.500	0.0046	0.500	0.0104	0.500	0.0104	0.500	0.0071	0.500	0.0104	0.500	0.0104

The lattice constants [experimental (*a*_ex_) and Rietveld refined (*a*_rv_)] were estimated by Nelson–Riley (N–R) extrapolation and Rietveld refinement method, respectively (Fig. 1c). The Al^3+^ (0.535 Å) ions were introduced by partially replacing Fe^3+^ (0.645 Å) ions in the octahedral site as a doping substance in the Ni–Zn–Co mixed ferrites. Depending upon the dopant's ionic radius and the cations distributions of the spinels, lattice parameters of the spinels show a decreasing trend with compositions of *x* = 0 − *x* = 0.12 (Fig. 1c).^1^ In addition, the lattice parameters and particle size of ferrite samples declined due to cation/anion vacancies, finite-size effect, lattice stress, *etc.*^12^

### 3.2 Porosities and densities

Bulk and X-ray densities play a crucial role in governing the polycrystalline ferrites' properties.^28,29^ The effect of Al^3+^ ions on the X-ray densities, bulk densities, and porosities is presented in Table 2. According to Jahan *et al.* 2021(1), grain size shows in Table 2. During synthesis, pores create inside the crystal structures of the samples. Hence, the compositions with *x* = 0 to *x* = 0.12 show less bulk densities than the theoretical (X-ray) densities (Table 2).^28^ The X-ray densities show a slight declination with Al contents *x* because Al^3+^ ions (26.98 g mol^−1^) have less molecular weight than the cations Fe^3+^ (55.84 g mol^−1^).^29,30^

**Table 2:** Table for compositions, X-ray density (*ρ*_x_ in g cm^−3^), bulk density (*ρ*_B_ in g cm^−3^), porosity (*P* in %), grain size (*D* in μm) of Al substituted Ni–Zn–Co ferrites

Compositions	X-ray density *ρ*_x_ (g cm^−3^)	Bulk Density, *ρ*_B_ (g cm^−3^)	Porosity, *P* (%)	Grain size, *D* (μm)
Ni_0.4_Zn_0.35_Co_0.25_Fe_2_O_4_	5.45	5.2	4.50	0.55
Ni_0.4_Zn_0.35_Co_0.25_Fe_1.98_Al_0.02_O_4_	5.43	5.18	4.64	0.47
Ni_0.4_Zn_0.35_Co_0.25_Fe_1.95_Al_0.05_O_4_	5.41	5.07	6.29	0.43
Ni_0.4_Zn_0.35_Co_0.25_Fe_1.93_Al_0.07_O_4_	5.40	5.09	5.72	0.45
Ni_0.4_Zn_0.35_Co_0.25_Fe_1.9_Al_0.1_O_4_	5.39	5.06	6.08	0.42
Ni_0.4_Zn_0.35_Co_0.25_Fe_1.88_Al_0.12_O_4_	5.38	4.98	7.37	0.38

Porosities, *P* (%), of the *x* = 0 − *x* = 0.12 samples can explain through the intragranular porosity (*P*_intra_) and intergranular porosity (*P*_inter_).^31^ During sintering, the thermal energy causes a force that pushes the grain boundaries to grow over pores. Thus the pore volume decreases and densifies the material.^24^ Sintering temperature (1200 °C) for the prepared ferrites remains the same throughout the studies; hence, intragranular porosities significantly have no effects on the compositions.^29,31^ During sintering inside the ferrites, intergranular porosities occur because of irregular particles.^1,28^ Besides, intergranular pores can change with the grain boundaries related to the grain growth (Table 2),^1^ demanding that the pores move together and merge, and a different transport mechanism indicates.^28^ Thus, an inverse relationship follows between the compositions' porosities and bulk densities.^28^ Furthermore, the gaseous oxygen flows through the pores, and the cations can diffuse around the pores. Due to more cations vacancies with reduced oxygen, the studied ferrites' porosities increase with Al concentrations *x*.^28^

### 3.3 TEM analysis

**Fig. 2:**
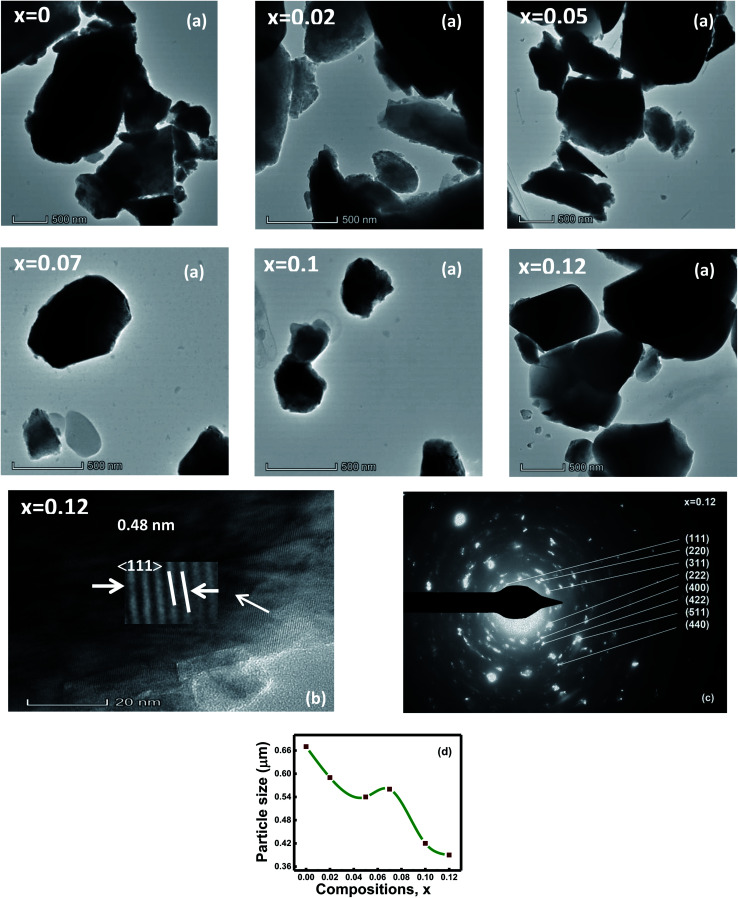
(a) TEM micrographs of all samples, (b) high-resolution transmission electron microscopy (HR-TEM) image of *x* = 0.12 sample, (c) selected area electron diffraction (SAED) pattern of *x* = 0.12 sample, and (d) variation of particle size (μm) with compositions *x* of the Ni_0.4_Zn_0.35_Co_0.25_Fe_(2−*x*)_Al_*x*_O_4_ (*x* = 0, *x* = 0.02, *x* = 0.05, *x* = 0.07, *x* = 0.1, and *x* = 0.12) spinel ferrites.

Fig. 2a shows the TEM micrographs and discloses that particles are irregular, and agglomerated. The average particle size estimated through TEM images indicates a decreasing trend with the composition that ranges from 0.67 to 0.39 μm (Fig. 2d). Thin areas of Al–O–Fe/Al–O–Al were generated during the particle growth with aluminum contents *x* that hindered further particle growth.^1,32^ Hence, particle size shows a decreasing trend with compositions. The agglomerated particles, on some level, are indicated due to the inter-magnetic interface between the particles.^33^ Besides, a reduction in surface energy is another cause of particle agglomeration.^34^

The HR-TEM micrograph discloses an extremely ordered lattice fringes with the *d*-spacings of 0.25, 0.48, 0.29, and 0.48 nm estimated by ImageJ software, demonstrating the (311), (111), (220), and (111) planes for the compositions with Al content *x* = 0, 0.02, 0.05, 0.07, 0.1, and 0.12, correspondingly. These *d*-spacing values are well-matched with the XRD analysis. The lattice images show equally spaced lattice rows suggesting that the areas are well-crystalline and free from any lattice defects.^33^Fig. 2b represents the HR-TEM micrograph of composition with *x* = 0.12.

The selected area (electron) diffraction (SAED) pattern of the ferrite samples displays the bright or dark bands due to the beams of light that are in phase or out of phase with one another.^35^ The SAED pattern of the composition with *x* = 0.12 is displayed in Fig. 2c. The diffraction rings of the SAED patterns are represented according to the space group *Fd*3*m* of the spinel-cubic lattice.

### 3.4 UV-vis absorption spectra studies

**Fig. 3:**
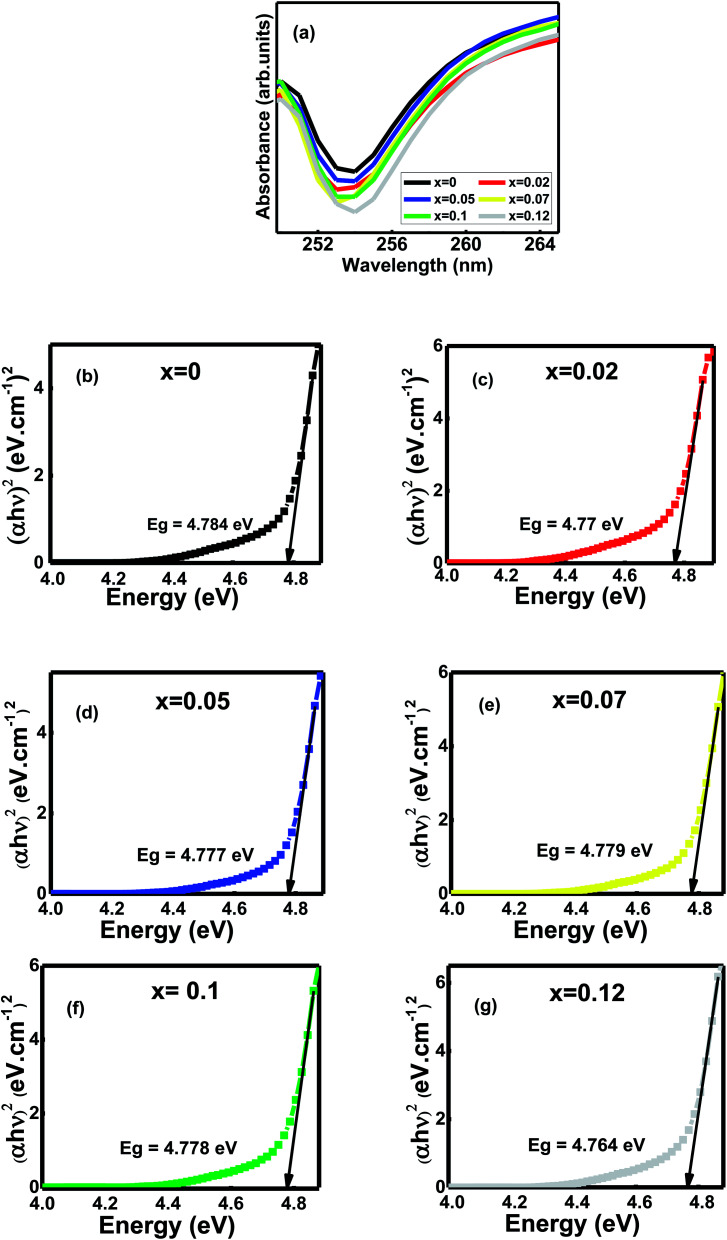
(a) UV-vis absorbance patterns at room temperature of Ni_0.4_Zn_0.35_Co_0.25_Fe_(2−*x*)_Al_*x*_O_4_ Ni_0.4_Zn_0.35_Co_0.25_Fe_(2−*x*)_Al_*x*_O_4_ (0 ≤ *x* ≤ 0.12) (b–g) Tauc plots of the compositions, *x*.

The absorption spectroscopy (UV-Vis) is one of the most important devices to measure the optical band gap energies (*E*_g_ in eV) of semiconductor materials like Al substituted Ni–Zn–Co mixed spinel ferrites. The ultraviolet-visible ray's absorption spectra were recorded in the wavelength range from 190 nm to 1100 nm at room temperature, but 249 nm to 265 nm is displayed (Fig. 3a). Hence, absorption peaks indicate that the photons are absorbed in the UV region around 253 nm. The electrons' photo-excitation between the valence and conduction bands or 3d^5^-3d^4^4s^1^ transition occurs for Fe^3+^ ions inside the studied mixed ferrites.^12^ However, both the redshift and blueshift are observed for the samples. The probable reason is the changes in their morphologies, grain size, carrier concentrations, surface microstructures, tiny amounts of impurities, lattice strain, and oxygen vacancy.^12^

The optical band gap (*E*_g_) is the most significant material feature suitable for optoelectronic applications.^12^ The samples' optical energy band gaps were calculated from the Tauc's plots using the UV-Vis absorbance spectral data (Fig. 3b–g). Energy band gaps range from 4.784–4.764 eV. The slight blue shift and redshifts observe as the grain size decreases from 0.55–0.38 μm with Al contents *x* (Table 2).^1^ The main reason for bandgap energies variation is the grains' defects and sub-bandgap energy levels generated during sintering.^12^ Moreover, annealing temperature influences the concentration of oxygen vacancies inside the samples.^12^ It creates trapped exciton states that form a series of metastable energy levels within the energy gap, resulting in the redshift/blueshift of the optical bandgap that varies the absorption band's in the wavelength region around 253 nm.^12^

### 3.5 Electrical properties analysis

#### 3.5.1 Study of permittivities and ac conductivities

**Fig. 4:**
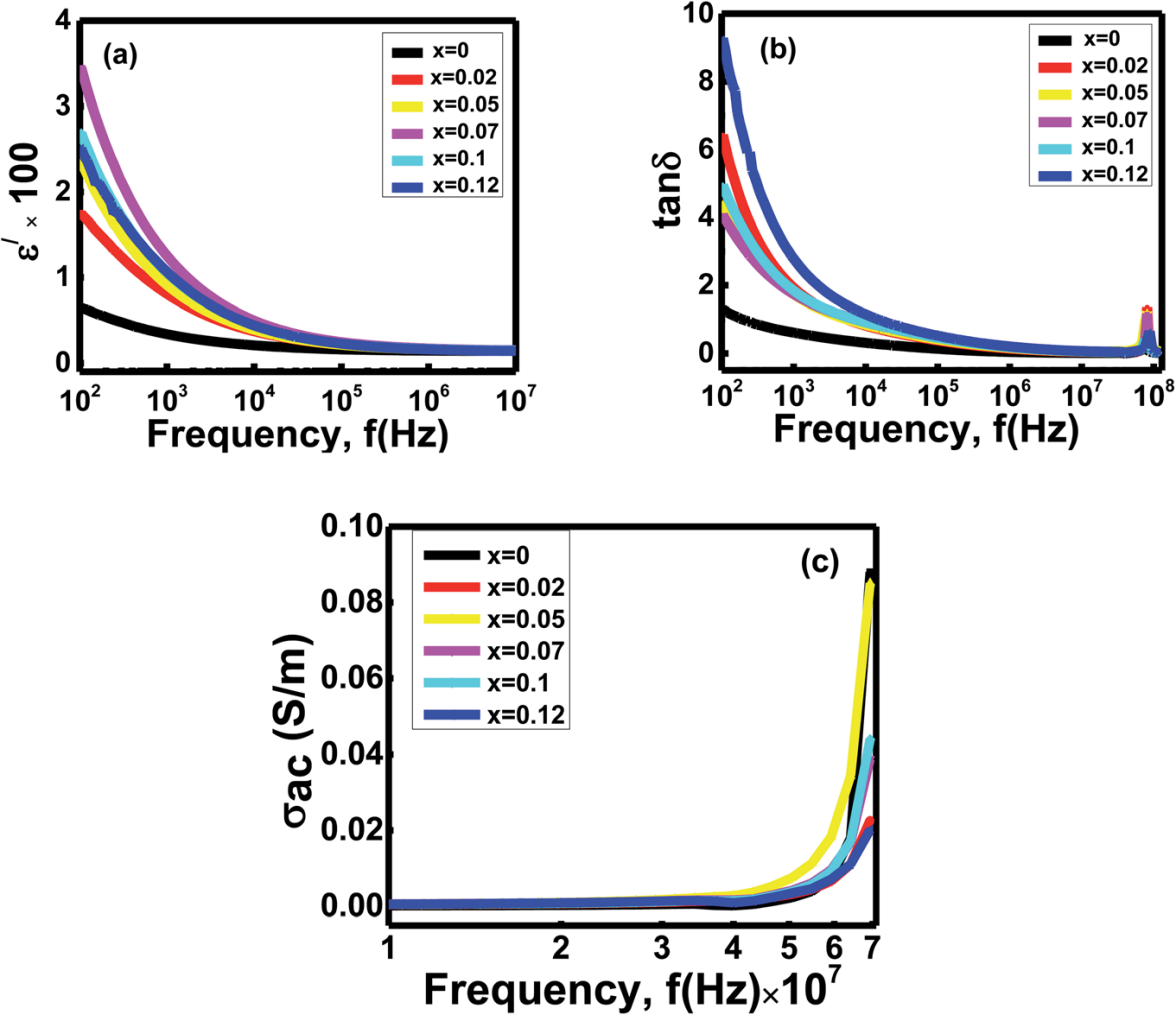
Variation of (a) dielectric constants (*ε*^/^) (b) loss tangent (tan *δ*) and (c) ac conductivity with frequency (*f* in Hz) of Ni_0.4_Zn_0.35_Co_0.25_Fe_(2−*x*)_Al_*x*_O_4_ (0 ≤ *x* ≤ 0.12) ferrites.

The frequency-dependent dielectric permittivity (*ε*^/^) and loss tangent of the Ni_0.4_Zn_0.35_Co_0.25_Fe_(2−*x*)_Al_*x*_O_4_ (0 ≤ *x* ≤ 0.12) ferrites examined at room temperature in the frequency range 10^2^ to 10^7^ Hz (Fig. 4a and b). The graph interprets that the dielectric constants (*ε*^/^) of the spinels of *x* = 0 − *x* = 0.12 give a dispersion at a low-frequency zone and gradually decrease with increasing frequencies (Fig. 4a). Furthermore, the dielectric constants of the studied ferrites behave almost independently in the high-frequency zone.

Koop's two-layer model and Maxwell–Wagner polarization theory explain the variation *ε*^/^ with frequency.^36^ It assumes electrons' hoping occurs between cations (Fe^3+^ and Co^2+^ ions, *etc.*) and O_2_ in a network that creates interactions.^36,37^ But when the electrons try to cross the insulating barriers (grain boundaries), electrons face an obstacle and cannot move from one grain to another. As a result, the trapped electrons pile up across the grain boundaries, and space charge polarization happens inside the studied ferrites' grains.^36,37^ Moreover, during sintering, there is a possibility of creating oxygen ions vacancies and free charge carriers inside the voids. Later they also take part in space charge polarization.^38^ At low frequency, the electrons get enough time to move across the grains by following the ac field and can participate in space charge polarization. Due to the rapid swing of the ac field at high frequencies, the electrons try to change their direction too quickly. Hence, the charge carriers cannot follow the area's path because of insufficient time. They cannot accumulate across the grain boundaries, and space charge polarization disappears inside the grains. Therefore, as frequency increases, the polarization gradually reduces and is independent in the high-frequency range.^36,38^

There is a dispersion of dielectric constants for the *x* = 0 to *x* = 0.12 samples at a low-frequency region. The grain boundaries' interfacial areas alter due to the differences in grain sizes with Al concentrations *x* (Table 2).^36^ Hence, the accumulations of charge carriers across the grain boundaries vary with compositions *x*. Thus, the space charge polarization shows slight deviations as Al^3+^ ions doping *x* carry on,^37^ and dispersion occurs for the samples' dielectric constants at the low-frequencies zone.

The frequency-dependent loss tangent graph (Fig. 4b) follows the same trend as the dielectric constants *vs.* frequency graph (Fig. 4a). The dielectric loss tangents for the samples *x* decline with increasing frequencies but reach a small peak in the high-frequency range around 7 × 10^7^ Hz.

Relaxation and resistive loss are the two mechanisms responsible for showing this dielectric loss inside the spinels. During the resistive loss mechanism, the charge carriers take energy from the materials due to the hoping, which occurs at low frequencies. But as frequency increases, the charge carriers do not get enough time to absorb energy from the ac field and accumulate across the grain walls. As a consequence, the loss minimizes. Significant resistive losses are observed at low frequencies but reduced at high frequencies. At high frequencies, the relaxations of dipoles waste energy inside the grains of the studied ferrites. Hence, relaxation loss becomes predominant in a high-frequency zone.^36^ During the sintering process, charge defect dipoles form because of the rearrangements of cations states. The small peaks of loss tangent are observed at high frequency because the hopping electron's frequency gets the same value as the applied field frequency. The spectra' peaks of the loss tangents identify the ferrite samples' resonance at the high frequencies around 9 × 10^7^ Hz.^36,38^ Due to different electronic configurations and grain sizes of the compositions *x*, the carriers' mobility fluctuates with the applied fields. Hence, dielectric loss values scatter at the low-frequency zone, and dispersion occurs for the samples *x*.^38^

Variation of ac conductivities (*σ*_ac_ = *ωε*_0_*ε*^//^)^39^ with frequencies of the studied ferrites are given in Fig. 4c. All the compositions exhibit almost flat plateaus until 10^7^ Hz for conduction. After that, sharp changes occur in conductivities with dispersion at 10^7^ to 10^8^ Hz.

The increase in the ferrites' ac conductivity with frequencies is described through Koop's two-layer model and the space charge. Ferrites materials have high conducting grains separated by grain boundaries having a resistive nature.^40^ The grains are highly conductive at high frequencies but exhibit low conductivity at a low frequency depending on electrons' hopping and space charge polarization.^41^ When the frequency of the applied field rises, the grains become more lively by increasing the hopping between ions, enhancing the hopping frequency.^38^ Moreover, the applied frequency's pumping forces support the charge carriers to transfer between the different localized states and release the trapped charges from the other trapping centers. The electrons and charge carriers contribute to the conduction mechanism triggered by valence exchange between the cations present inside the sublattices.^38,42^ Thus a sharp enhancement in ac conductivities is observed for all the compositions. However, these hopping charge carriers strongly correlate to the conduction mechanism and dielectric behavior. As the applied field frequency increases, the dielectric constant decreases and obtains a constant value because the polarization could not follow the ac fields fluctuations. So the total polarization decreases and enhances the conductivities for all the ferrite samples.^41^

But in the low-frequency region, the ac field does not provide adequate energy to the carriers to overcome these insulating barriers. The samples behave like semiconductors, and a flat plateau is observed.^43^ A dispersion of conductions is occurred at a high-frequency zone because of several microstructural features and band gaps (Fig. 3c) of the studied ferrites. Besides, oxygen deficiency plays a vital role in charge carriers' mobility.^44^ Conductivity increases with the oxygen vacancy concentration.^44^ The changing of particle sizes with compositions, *x*, changes the number of grain boundaries that vary the electron mobility from one grain to another.^44^ Due to the grain boundary's conductive property, the samples' oxygen vacancy concentration may influence the conductivity. Hence, the hoping of electrons plays a significant role in increasing the conductivity of the studied ferrites.^44^

#### 3.5.2 Impedance spectra study

**Fig. 5:**
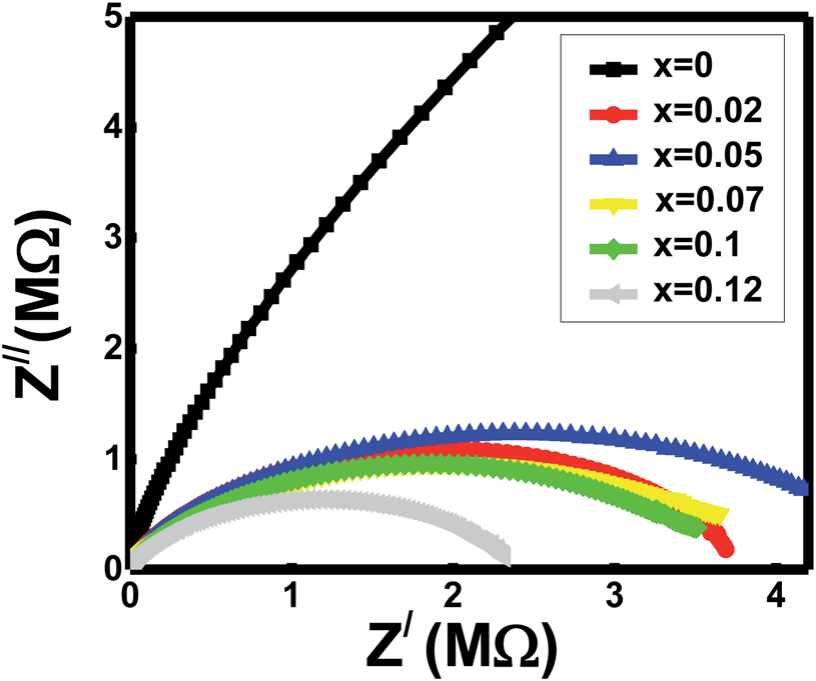
Cole–Cole plot of impedance of the Al-substituted Ni–Zn–Co ferrites.

All the studied ferrites with *x* = 0 − *x* = 0.12 in the Cole–Cole impedance plot (Nyquist plot) show the semicircle patterns (Fig. 5). The actions interpret the conduction's main contributions inside the lattices because of the charge carrier's mobility through the grain boundary volume.^43^ The grains are assumed to be the conducting plates, and the grain boundaries are the two insulating plates on two sides of the grains. The grain boundary is a general planer defect that splits regions of different crystalline orientations (grains) within the polycrystalline material, creating obstacles to electrical conductivity. These inherent resistive properties of grain boundaries obtain by depletion of oxygen vacancies by forming a space charge zone in the vicinity of the grain-boundary core, leading to high resistance to oxygen-cationic transport through them.^45^ Space-charge polarization plays a crucial role in controlling the conduction across the grain boundary and is named grain boundary resistance (*R*_gb_).^45^ Space charge polarization diminishes significantly at the high-frequency zone, and the hoping mechanism occurs inside the conductive grains. Due to free charges collisions and an insignificant amount of space charge polarization, grain offers some resistance named grain resistance (*R*_g_).

Cole–Cole plot represents the semicircle at the axis of the real part (*Z*^/^) in the low-frequency zone (right side) gives the values of total resistance (*R*_T_ = *R*_g_ + *R*_gb_) for spinels *x*. All the ferrites show high resistance values around 10^7^ to 10^8^ ohm. The samples' grain resistance (*R*_g_) values were taken from the semicircle intersection with the real axis at the high-frequency zone (left side). The obtained values of *R*_g_ for the ferrites are within 50 ohms. Thus, *R*_gb_ calculates the relationship between the resistances, *R*_gb_ = *R*_T_ − *R*_g_. It observes from the spectra that the grain resistances are almost negligible in respect of total resistances. Hence, the grain boundary resistances (*R*_gb_) for the compositions *x* are nearly equal to total resistance values, *R*_T_. The grain boundary resistances (*R*_gb_) contribute to the present studied samples' conductions. It also reveals that the semicircles represented mainly the grain boundaries' characteristic behaviours.^43,46^

#### 3.5.3 Electric modulus analysis

**Fig. 6:**
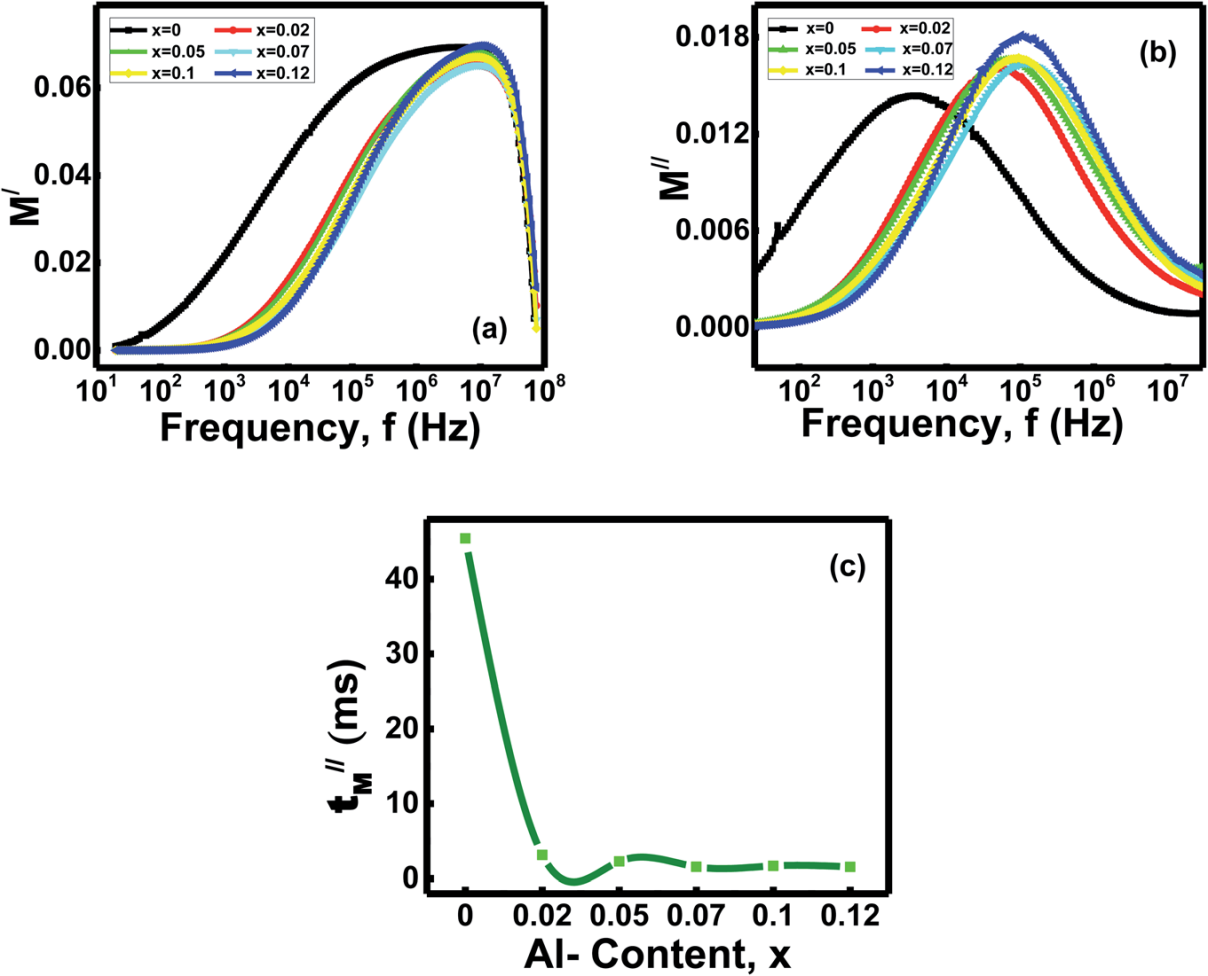
(a) Variation of real part complex electrical modulus with frequency (b) variation of imaginary part complex electrical modulus with frequency and (c) relaxation time (*τ*^//^_M_) with compositions of Ni_0.4_Zn_0.35_Co_0.25_Fe_(2−*x*)_Al_*x*_O_4_ (0 ≤ *x* ≤ 0.12) ferrites.

The frequency dependence behaviors of real part electric modulus [*M*^/^(*ω*) = *ε*^/^/(*ε*^/2^ + *ε*^//2^)] for the studied ferrites are plotted in the Fig. 6a.^47^ Initially, at low frequencies, the spinels' spectra show shallow values of *M*^/^(*ω*). Besides, *M*^/^(*ω*) values gradually increase with frequencies and show maximum peaks at around 10^7^ Hz for all the compositions. The electric modulus's real part gradually decreases to low values with frequencies around 10^8^ Hz. *M*^/^(*ω*)'s tendency to reach the maximum for the samples confirms the voids' relaxation mechanism in the high-frequency zone. Hence, this quick-rising of *M*^/^(*ω*) at a high frequency of around 10^7^ Hz indicating short-range mobility of charge carriers, happens during the conduction. Whereas at the low-frequency zone, the small values of *M*^/^(*ω*) indicate that the negligible amount of electrode polarization arises inside the grains of the tested compositions *x*.^43^

Fig. 6b showed the variation of imaginary parts of electrical modulus [*M*^//^(*ω*) = *ε*^//^/(*ε*^/2^ + *ε*^//2^)] with frequencies for the ferrites *x*.^47^ It observes *M*^//^(*ω*) yields low values at lower frequencies, but when the frequency reaches the range around 10^4^ to 10^5^ Hz, *M*^//^(*ω*) shows broad maxima for the samples x. Later on, the peaks of *M*^//^(*ω*) for the ferrites *x* gradually decrease to low values with other higher frequencies. The charge carriers can move long distances inside the grains at low frequencies and show very high capacitance values. As a result, the ferrite samples show very low *M*^//^(*ω*) values. The carriers are confined inside the potential wells and do not follow the ac fields at the high-frequency zone. Hence, the charges move in short-range distances.^43^ The ferrites at high frequencies exhibit wide and asymmetry peaks on both sides of the maxima.

Variation of relaxation times (*τ*^//^_M_ = 1/2π*f*_M_^//^) with compositions *x* are shown in the Fig. 6c.^48^ The relaxation time for the *x* = 0 composition is 45.45 μs but drops down from 3.13–1.54 μs for the samples with *x* = 0.02 − *x* = 0.12. Thus, it identifies that the Al-doped ferrites have less relaxation time than ferrite without Al-doping.

### 3.6 Study of magnetic parameters

#### 3.6.1 Study of Curie's temperature

**Fig. 7:**
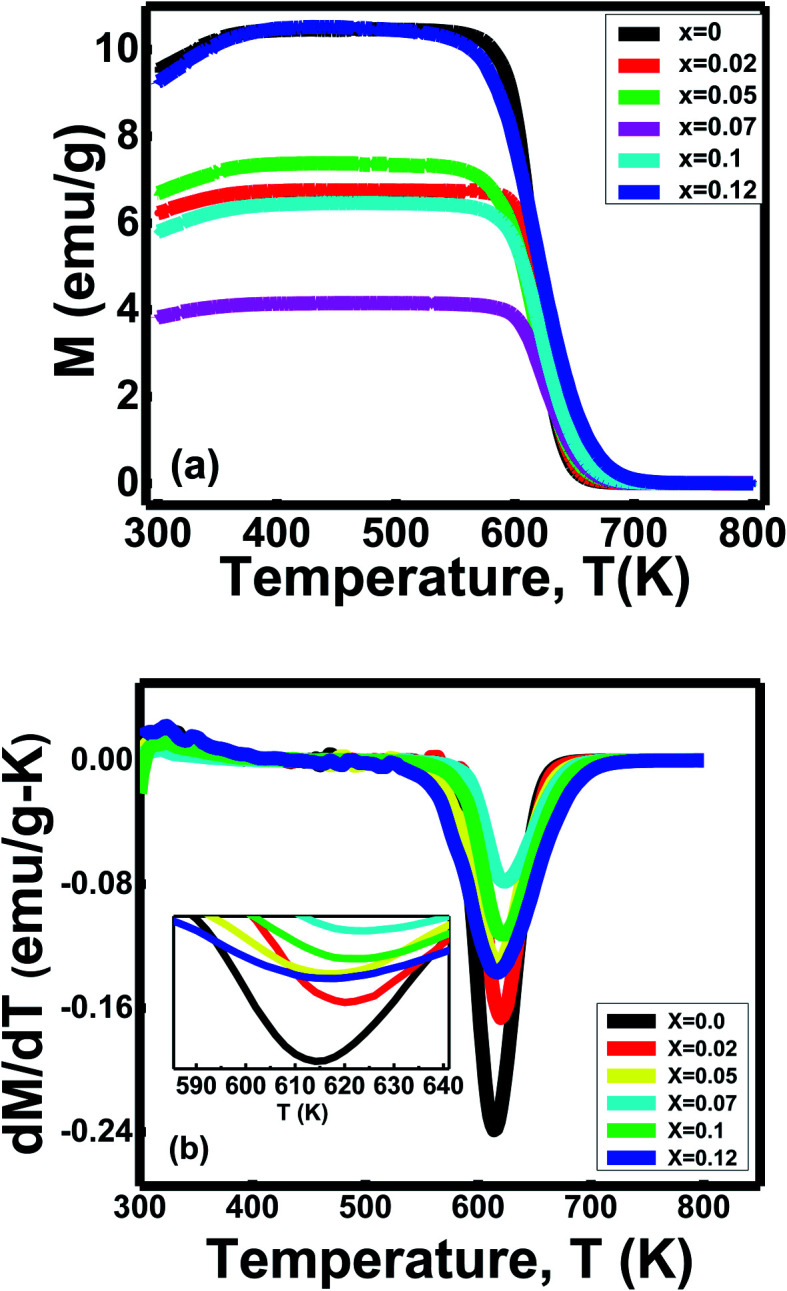
(a) Variation of magnetization with temperature, (b) d*M*/d*T vs. T* graph of Ni_0.4_Zn_0.35_Co_0.25_Fe_(2−*x*)_Al_*x*_O_4_ (0 ≤ *x* ≤ 0.12) ferrites.

The behavior of magnetizations (*M* in emu g^−1^) with temperature (300 K to 800 K) was observed for ferrites with *x* = 0 to *x* = 0.12 under an applied magnetic field (100 Oe (Fig. 7a). Ferromagnetic-paramagnetic (FM-PM) phase transitions mark Curie's temperature *T*_c_ in the magnetization *vs.* temperature graph. The Curie temperature (*T*_C_) is the critical temperature above which a ferromagnetic material loses its residual magnetism and becomes paramagnetic. Curie's temperatures (*T*_c_ in K) determine through the temperatures at which d*M*/d*T* showed a minimum in the d*M*/d*T vs. T* graph for the compositions *x* (Fig. 7b).^49^

Ferrites' Curie temperature (*T*_c_) fundamentally depends on A–B exchange interaction.^12^ The substitution of Al^3+^ ions reduces the Curie temperature due to the distortion of some ferromagnetic ordering inside the systems.^49^ The electronic configuration 2p^6^ of nonmagnetic ion Al^3+^ influences the ferromagnetic double exchange between Fe^3+^–O^2–^–Fe^3+^.^49^ Therefore, a new coupling between Al^3+^–O^2–^–Al^3+^ generates without contributing to the magnetic interactions.^49^ Hence, Curies temperature decreases for the sample with *x* = 0.05, *x* = 0.1 and *x* = 0.12. Moreover, the oxygen deficiency inside the spinels plays a significant role in the magnetic structures due to the changing valence states.^50^ It also diluted the superexchange interaction between cations through intervening oxygen, reducing Curie's temperature.^50^

Furthermore, Curie temperature is also influenced by grain size.^12^ The variation of Curie temperature with grain sizes is due to a change in cation arrangement between the A site and B site of the spinel lattice based on the strength of the exchange interactions.^12^ The decrease in lattice parameters caused increased A–B exchange interaction.^12^ As the exchange interaction is relatively strong, consistently higher energy is required to disorient the moment representing higher *T*_C_ values with decreasing particle size for the sample with *x* = 0.02 and *x* = 0.07.^12^ Therefore, variation in Curie's temperature (*T*_c_) indicates the formation of new ferrites with an overall enhancement of phase transition temperature (*T*_c_).

#### 3.6.2 Permeability analysis

**Fig. 8:**
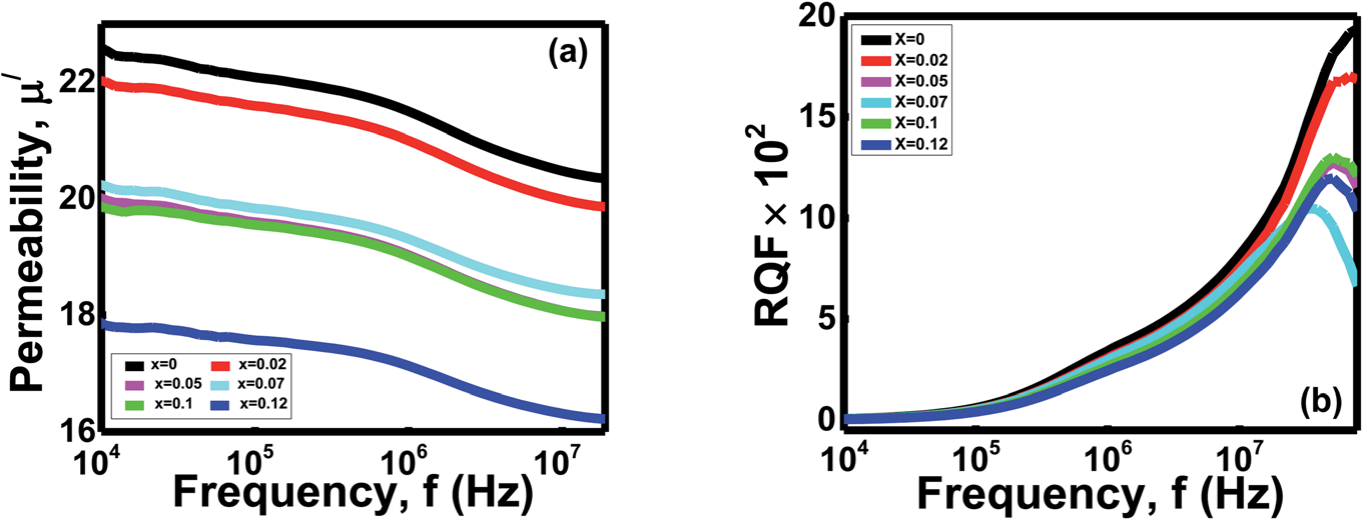
Variation of (a) real part of permeability (*μ*^/^) with frequency (b) RQF with frequency of Ni_0.4_Zn_0.35_Co_0.25_Fe_(2−*x*)_Al_*x*_O_4_ (0 ≤ *x* ≤ 0.12) ferrites.

The frequency-dependent permeability of the Ni_0.4_Zn_0.35_Co_0.25_Fe_(2−*x*)_Al_*x*_O_4_ (0 ≤ *x* ≤ 0.12) ferrites recorded at room temperature. The influences of frequencies on the real part permeability (*μ*^/^) are shown in Fig. 8a. All the ferrites *x* give almost a constant value until the frequency 10^6^ Hz, and after that, the real part permeability drops down to a low value at a high-frequency zone. The flat plateaus of *μ*^/^ under a wide range of frequencies until 10^6^ Hz show the prepared spinels' quality and stability.^51^

The *μ*_i_ of ferrites are affected by intrinsic features like favoured site residence and extrinsic properties such as density, porosity, and grain size.^10,52^ Polycrystalline ferrites' permeability is influenced by the superposition of the spin rotation and domain wall motion during synthesis.^52^ As *μ*_i_ is delicate with some factors, it is still exciting to conclude a specific conclusion for changing *μ*_i_ with concentration. Permeability is proportional to grain size for normal grain growth in ferrites.^43^ Variations in grain size of the samples can cause a change in every grain's domain walls number.^43^ As the domain wall movement controls the *μ*_i_, any variation in the domain walls number manipulates *μ*_i_^/^.^52^ Moreover, the declining trend of *μ*_i_/demonstrated that the ionic radii of Al^3+^ might be larger than the lattice of spinel, so Al^3+^ finds the struggle to enter the spinel lattice. Hence, significant amounts of Al^3+^ resided at grain boundaries and formed a thin structure of Al–O–Al.^1,52^ This narrow structure around the magnetic domain hinders the grain boundary mobility and grain growth for the Ni_0.4_Zn_0.35_Co_0.25_Fe_(2−*x*)_Al_*x*_O_4_ (0 ≤ *x* ≤ 0.12) ferrites.^51^ Besides, *μ*_i_^/^ displays a reducing trend with increasing frequency due to nonmagnetic impurities between grains and intragranular pores.^52^ It functions as a pinning center at higher frequencies and obstructs spin and domain wall motion.^52^ In the current study, density is inversely proportional to the porosity (Table 1), and bulk density is proportional to particle size. The correlation between the ferrite samples' porosity, bulk density, and particle size *x* indicates the highest *μ*_i_^/^ obtain with the lowest porosity and maximum particle size.

The ferrites' relative quality factor (RQF) shows that the peak at the high-frequency range is around 10^7^ to 10^8^ Hz and then gradually decreases to low values (Fig. 8b). The ferrite with *x* = 0 shows the highest RQF than the samples with *x* = 0.02 − *x* = 0.12. The porosity and thin Al–O–Al area act as obstacles and pining centers against the studied compositions' domain wall motion.^1,51^ Thus, energy dissipates as a hysteresis loss due to overcoming these barriers to continue the domain wall motion,^51^ and different values of RQF obtain.

## 4. Conclusions

In the current study, Al^3+^-doped Ni–Zn–Co ferrite samples were successfully synthesized through the conventional ceramic technique. X-ray diffraction analysis evidenced the formation of the single-phase cubic spinel structures with phase group *Fd*3̄*m* for all compositions. As demonstrated by TEM examinations, average particle sizes ranged from 0.67 to 0.39 μm. An insignificant conductivity until the frequency of 10^7^ Hz and a sudden rise hereafter exhibited that the samples were very resistive. Relaxation times were reduced to a low value from 45.45 μs to 1.54 μs with increasing doping contents x, implying that the Al^3+^ played a vital role in the change carriers' mobility. Curie temperature (*T*_c_) was 615 K–623 K, which showed an overall enhancement. Permeability was directly proportional to the grain size demonstrated for all samples. The undoped composition corresponds to the highest *μ*_i_^/^ obtained with the lowest porosity and maximum particle size. The real part of permeability (*μ*_i_^/^) was independent of frequency until 10^6^ to 10^7^ Hz with relative quality factors of around 10^3^. Al^3+^ substitution implemented a central role in tuning the Ni–Zn–Co system's electromagnetic properties that can be promising materials for high-frequency devices.

## Author contributions

Nusrat Jahan: conceptualization, methodology, software, validation, formal analysis, investigation, resources, data curation, roles/writing - original draft, visualization, writing-reviewing, and editing, M. N. I. Khan: supervision, writing-reviewing, and editing, M. R. Hasan: investigation, M. S. Bashar: investigation, A. Islam: investigation, M. K. Alam: investigation, M. A. Hakim: writing-reviewing, and editing, J. I. Khandaker: supervision, writing-reviewing, and editing.

## Conflicts of interest

The authors announce that they have no known competing financial interests or personal relationships that could have appeared to influence the work reported in this paper.

## Supplementary Material

## References

[cit1] Jahan N., Khandaker J. I., Liba S. I., Hoque S. M., Khan M. N. I. (2021). Structural analysis through cations distributions of diamagnetic Al^3+^ ions substituted Ni-Zn-Co ferrites. J. Alloy. Compd..

[cit2] Qindeel R., Alonizan N. H., Alghamdi E. A., Awad M. A. (2021). Synthesis and characterization of spinel ferrites for microwave devices. J. Sol-Gel Sci. Technol..

[cit3] Bromho T. K., Ibrahim K., Kabir H., Rahman M. M., Hasan K., Ferdous T., Taha H., Altarawneh M., Jiang Z. (2018). Understanding the impacts of Al^3+^ substitutions on the enhancement of magnetic, dielectric and electrical behaviors of ceramic processed nickel-zinc mixed ferrites: FTIR assisted studies. Mater. Res. Bull..

[cit4] Tiwari P., Kane S. N., Mazaleyrat F., Deshpande U. P. (2020). Composition assisted tuning properties of CoCr_*x*_Fe_2−*x*_O_4_ spinel nano ferrites. Mater. Today: Proc..

[cit5] Bharadwaj P. S. J., Prasad Gannavarapu K., Sai Kollipara V., Babu Dandamudi R. (2021). Study of magneto-supercapacitance properties of nickel cobalt ferrite-activated carbon composite. J. Energy Storage.

[cit6] Tatarchuk T., Shyichukc A., Trawczyńskac I., Yaremiyd I., Pędziwiatre A. T., Kurzydłoe P., Bogacze B. F., Gargula R. (2020). Spinel cobalt(II) ferrite-chromites as catalysts for H_2_O_2_ decomposition: synthesis, morphology, cation distribution and antistructure model of active centers formation. Ceram. Int..

[cit7] Tiwaria R., Dea M., Tewaria H. S., Ghoshalb S. K. (2020). Structural and magnetic properties of tailored NiFe_2_O_4_ nanostructures synthesized using auto-combustion method. Results Phys..

[cit8] Maksoud M. I. A. A., El-Ghandour A., El-Sayyad G. S., Fahim R. A., El-Hanbaly A. H., Bekhit M., Abdel-Khalek E. K., El-Bahnasawy H. H., Abd Elkodous M., Ashour A. H., Awed A. S. (2020). Unveiling the Effect of Zn^2+^ Substitution in Enrichment of Structural, Magnetic, and Dielectric Properties of Cobalt Ferrite. J. Inorg. Organomet. Polym. Mater..

[cit9] Derakhshani M., Taheri-Nassaj E., Jazirehpour M., Masoudpanah S. M. (2021). Structural, magnetic, and gigahertz-range electromagnetic wave absorption properties of bulk Ni–Zn ferrite. Sci. Rep..

[cit10] Vinnik D. A., Zhivulin V. E., Sherstyuk D. P., Starikov A. Y., Zezyulina P. A., Gudkova S. A., Zherebtsov D. A., Rozanov K. N., Trukhanov S. V., Astapovich K. A., Turchenko V. A., Sombra A. S. B., Zhou D., Jotania R. B., Singh C., Trukhanov A. V. (2021). Electromagnetic properties of zinc–nickel ferrites in the frequency range of 0.05–10 GHz. Mater. Today Chem..

[cit11] Stergiou C. (2016). Microstructure and Electromagnetic Properties of Ni-Zn-Co Ferrite up to 20 GHz. Adv. Mater. Sci. Eng..

[cit12] Massoudi J., Smari M., Nouri K., Dhahri E., Khirouni K., Bertaina S., Bessais L., Hlil E. K. (2020). Magnetic and spectroscopic properties of Ni–Zn–Al ferrite spinel: from the nanoscale to microscale. RSC Adv..

[cit13] Patil1 B. B., Pawar A. D., Bhosale D. B., Ghodake J. S., Thorat J. B., Shinde T. J. (2019). Effect of La3+ substitution on structural and magnetic parameters of Ni–Cu–Zn nano-ferrites. J. Nanostructure Chem..

[cit14] Abbasa Q., Murtazaa G., Muhammada N., Ishfaqa M., Iqbala H. M. T., Asadb A., Ashrafc G. A., Iqbald M. Z. (2020). Structural, dielectric and magnetic properties of (ZnFe_2_O_4_/polystyrene)nanocomposites synthesized by micro-emulsion technique. Ceram. Int..

[cit15] Patil1 B. A., Kounsalye J. S., Humbe A. V., Kokate R. D. (2021). Structural, magnetic, dielectric and hyperfine interaction studies of titanium (Ti^4+^)-substituted nickel ferrite (Ni_1+*x*_Ti_*x*_Fe_2−2*x*_O_4_) nanoparticles. J. Mater. Sci.: Mater. Electron..

[cit16] S Das P., Singh G. P. (2016). Structural, magnetic and dielectric study of Cu substituted NiZn ferrite nanorod. J. Magn. Magn. Mater..

[cit17] Dippong T., Levei E. A., Deac I. G., Neag E., Cadar O. (2020). Influence of Cu^2+^, Ni^2+^, and Zn^2+^ Ions Doping on the Structure, Morphology, and Magnetic Properties of Co-Ferrite Embedded in SiO_2_ Matrix Obtained by an Innovative Sol-Gel Route. J. Nanomater..

[cit18] Li J., Huan A. C. H., Wang L., Du Y., Feng D. (2000). Interface effects on magnetoresistance and magnetic-field-reduced Raman scattering in magnetite. Phys. Rev. B.

[cit19] Atif M., Asghar M., Nadeem M., Khalid W., Ali Z., Badshah S. (2018). Structural, magnetic and dielectric study of Cu substituted NiZn ferrite nanorod. J. Phys. Chem. Solids.

[cit20] Yaseneva P., Bowker M., Hutchings G. (2011). Structural and magnetic properties of Zn-substituted cobalt ferrites prepared by co-precipitation method. Phys. Chem. Chem. Phys..

[cit21] Li J., Zeng X., Xu Z. (2013). Partial cationic inversion-induced magnetic hardening of densely packed 23-nm-sized nanocrystallite-interacting nickel ferrite electrospun nanowires. Appl. Phys. Lett..

[cit22] Afzal A. M., Manzoor A., Khan M. Z., Khan M. F., Skindar A., Anwer U., Ahmad Z. (2019). Effect of Cobalt Substitution on Magnetic, Resistivity and Dielectric Properties of Nickel–Zinc Ferrite (Co_*x*_Ni_*x*−0.5_Zn_0.5_Fe_2_O_4_). J. Nanoelectron. Optoelectron..

[cit23] Kumar R. V., Anupama A. V., Kumar R., Choudhary H. K., Khopkar V. B., Aravind G. (2018). Cation distributions and magnetism of Al-substituted CoFe_2_O_4_ – NiFe_2_O_4_ solid solution synthesis by sol-gel auto-combustion method. Ceram. Int..

[cit24] Bharati V. A., Somvanshi S. B., Humbe A. V., Murumkar V. D., Sondur V. V., Jadhav K. M. (2020). Influence of trivalent Al-Cr co-substitution on the structural, morphological, and Mossbauer properties of nickel ferrite nanoparticles. J. Alloys Compd..

[cit25] Hossain M. D., Jamil A. T. M. K., Sarowar Hossain M., Ahmed S. J., Das H. N., Rashid R., Hakim M. A., Khan M. N. I. (2022). Investigation on structure, thermodynamic and multifunctional properties of Ni–Zn–Co ferrite for Gd^3+^ substitution. RSC Adv..

[cit26] Rodríguez-Carvajal J. (1993). Recent advances in magnetic structure determination by neutron powder diffraction. Phys. B.

[cit27] Datt G., Bishwas M. S., Raja M. M., Abhyankar A. C. (2016). Observation of magnetic anomalies in one-step solvothermally synthesized nickel–cobalt ferrite nanoparticles. Nanoscale.

[cit28] Akhter S., Paul D. P., Hakim M. A., Saha D. K., Al-Mamun M., Parveen A. (2011). Synthesis, Structural and Physical Properties of Cu_1–*x*_Zn_*x*_Fe_2_O_4_ Ferrites. JMSA.

[cit29] Hossain A. K. M. A., Rahman M. L. (2011). Enhancement of microstructure and initial Permeability due to Cu substitution in Ni_0.50−*x*_Cu_*x*_Zn_0.50_Fe_2_O_4_ ferrites. J. Magn. Magn. Mater..

[cit30] Somvanshi S. B., Khedkar M. angesh V., Kharat P. B., Jadhav K. M. (2020). Influential diamagnetic magnesium (Mg^2+^) ion substitution in nano-spinel zinc ferrite (ZnFe_2_O_4_): thermal, structural, spectral, optical and physisorption analysis. Ceram. Int..

[cit31] Raju M. K., Raju M. R., Babu K. R., Patnaik J. R. G., Samatha K. (2015). Structural Observations, Density and Porosity Studies of Cu Substituted Ni-Zn Ferrite through Standard Ceramic Technique. Chem. Sci. Trans..

[cit32] Kumar R. V., Anupama A. V., Kumar R., Choudhary H. K., Khopkar V. B., Aravind G. (2018). Cation distributions and magnetism of Al-substituted CoFe_2_O_4_ – NiFe_2_O_4_ solid solution synthesis by sol-gel auto-combustion method. Ceram. Int..

[cit33] Humbe A. V., Kounsalye J. S., Somvanshi S. B., Kumar A., Jadhav K. M. (2020). Cation distribution, magnetic and hyperfine interaction studies of Ni−Zn spinel ferrites: role of Jahn Teller ion (Cu^2+^) substitution. Mater. Adv..

[cit34] Shrestha S., Wang B., Dutta P. (2020). Nanoparticle processing:Understanding and controlling aggregation. Adv. Colloid Interface Sci..

[cit35] Vlazan P., Miron I., Sfirloaga P. (2015). Cobalt ferrite substituted with Mn: Synthesis method, characterization and magnetic properties. Ceram. Int..

[cit36] Duvuru H. B., Alla S. K., Shaw S. K., Meena S. S., Gupta N., Prasad B. B. V. S. V., Kothawale M. M., Kumar M. K., Prasad N. K. (2019). Magnetic and dielectric properties of Zn substituted cobalt oxide nanoparticles. Ceram. Int..

[cit37] Atif M., Idrees M., Nadeem M., Siddique M., Ashraf M. W. (2016). Investigate on structural, Dielectric, and impedance analysis of manganese substituted cobalt ferrite i.e., Co_1−*x*_Mn_*x*_Fe_2_O_4_ (0.0 ≤ *x* ≤ 0.4). RSC Adv..

[cit38] Kaur P., Chawla S. K., Meena S. S., Yusuf S. M., Narang S. B. (2016). Synthesis of Co-Zr doped nanocrystalline strontium hexaferrites by sol-gel auto- combustion route using sucrose as fuel and study of their structural, magnetic and electrical properties. Ceram. Int..

[cit39] Donta P., Katrapally V. K., Pendyala V. R. (2016). Dielectric Response of Ni_*x*_ Zn_1−*x*_Al Fe O_4_ Nanoferrites. NanoWorld J..

[cit40] Furhaj A. S., Muhammad K., Muhammad S. S., Asghar H. M. N. H. K., Sameen A., Ayesha P., Jalil U. R., Muhammad A. K., Zaheer A. G. (2019). Effects of bismuth on structural and dielectric properties of cobalt-cadmium spinel ferrites fabricated via micro-emulsion route. Phys. B.

[cit41] Darwish M. A., Trukhanov A. V., Senatov O. S., Morchenko A. T., Saafan S. A., Astapovich K. A., Trukhanov S. V., Trukhanova E. L., Pilyushkin A. A., Sombra A. S. B., Zhou D., Jotania R. B., Singh C. (2020). Investigation of AC-Measurements of Epoxy/Ferrite Composites. Nanomater.

[cit42] Hashim M., Hcini S., Kumar S., Shirsath S. E., Mohammed E. M., Kumar R. (2013). Structural, Dielectric, AC Conductivity, and Magnetic Properties of Cr^3+^ Substituted Ni–Mg Ferrite Nanoparticles. J. Nanoeng. Nanomanuf..

[cit43] Oumezzine E., Hcini S., Rhouma F. I. H., Oumezzine M. (2017). Frequency and temperature dependence of conductance, impedance, and electrical modulus studies of Ni_0.6_Cu_0.4_Fe_2_O_4_ spinel ferrite. J. Alloys Compd..

[cit44] Bouzayen M., Dhahri R., Benali A., Chaabouni S., Khirouni K., Costa B. F. O. (2021). Synthesis and investigation of oxygen deficiency effect on electric properties of La_0.75_Ba_0.10_Sr_0.15_FeO_2.875−*δ*_ (*δ* = 0.00, 0.125 and 0.25) ferrites. J. Mater. Sci.: Mater. Electron..

[cit45] Kim S. (2009). The intrinsic origin of the grain-boundary resistance in Sr-doped LaGaO_3_. Monatsh. Chem..

[cit46] Rahmouni H., Selmi A., Khirouni K., Kallel N. (2012). Chromium effects on the transport properties in La_0.7_Sr_0.3_Mn_1−*x*_Cr_*x*_O_3_. J. Alloys Compd..

[cit47] Bharathi K. K., Markandeyulu G., Ramana C. V. (2011). Impedance Spectroscopic Characterization of Sm and Ho Doped Ni Ferrites. J. Electrochem. Soc..

[cit48] Hossain M. D., Khan M. N. I., Naharb A., Ali M. A., Matin M. A., Hoque S. M., Hakim M. A., Jamil A. T. M. K. (2020). Tailoring the properties of Ni-Zn-Co ferrites by Gd^3+^ substitution. J. Magn. Magn. Mater..

[cit49] Baazaoui M., Farah Kh., Hosni F., Cheikhrouhou-Koubaa W., Oumezzine M. (2019). Magnetocaloric Effect and Electron Paramagnetic Resonance Study of Gallium-Doped La_0.65_Bi_0.05_Sr_0.3_Mn_1−*x*_Ga_*x*_O_3_ (*x* = 0 and 0.06) manganites. J. Low Temp. Phys..

[cit50] Sugiyama J., Atsumi T., Koiwai A., Sasaki T., Hioki T., Noda S., Kamegashira N. (1997). The effect of oxygen deficiency on the structural phase transition and electronic and magnetic properties of the spinel LiMn_2_O_4−*δ*_. J. Phys.: Condens. Matter.

[cit51] Ali M. A., Khan M. N. I., Chowdhury F.-U.-Z., Hossain M. M., Hossain A. K. M. A., Rashid R., Nahar A., Hoque S. M., Matin M. A., Uddin M. M. (2019). Yttrium-substituted Mg-Zn ferrites: correlation of physical properties with Yttrium content. J. Mater. Sci.: Mater. Electron..

[cit52] Rashid M. H., Hossain A. K. M. A. (2018). Structural, morphological and electromagnetic properties of Sc^3+^ doped Ni-Cu-Zn ferrites. Results Phys..

